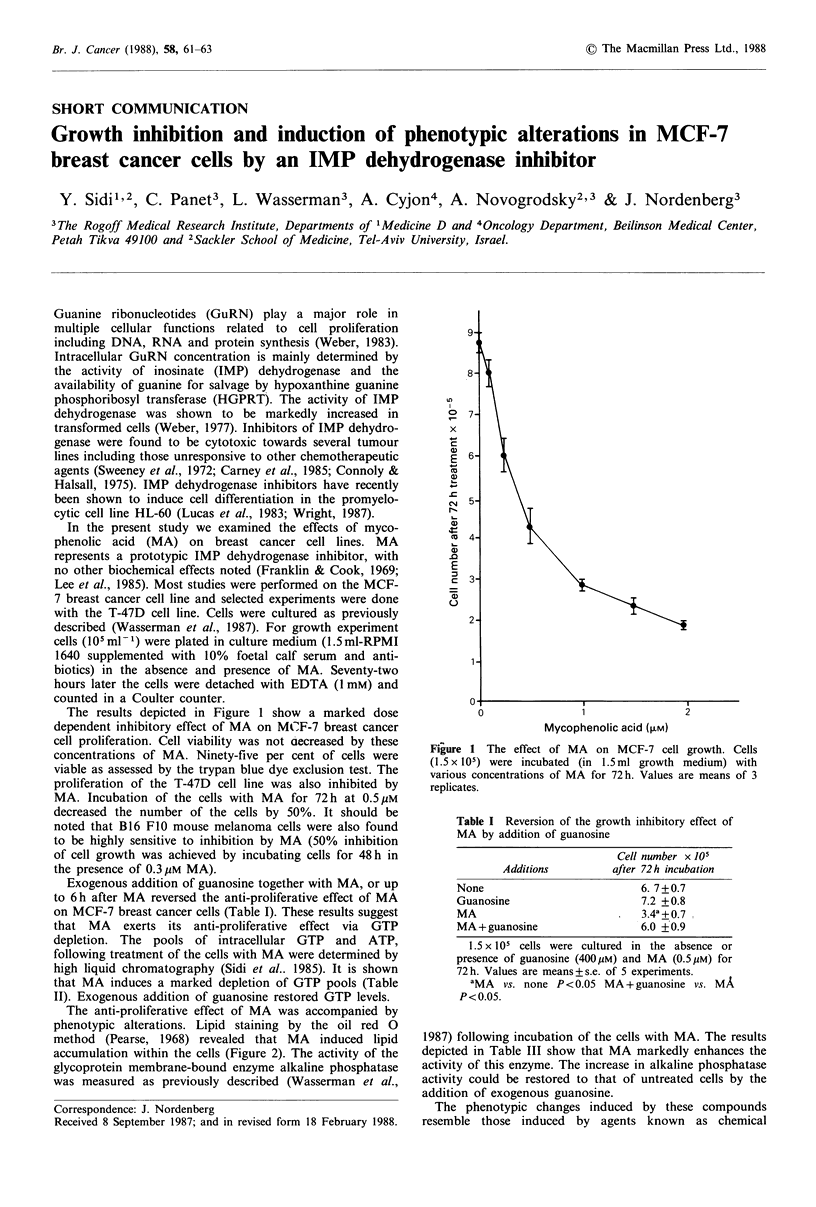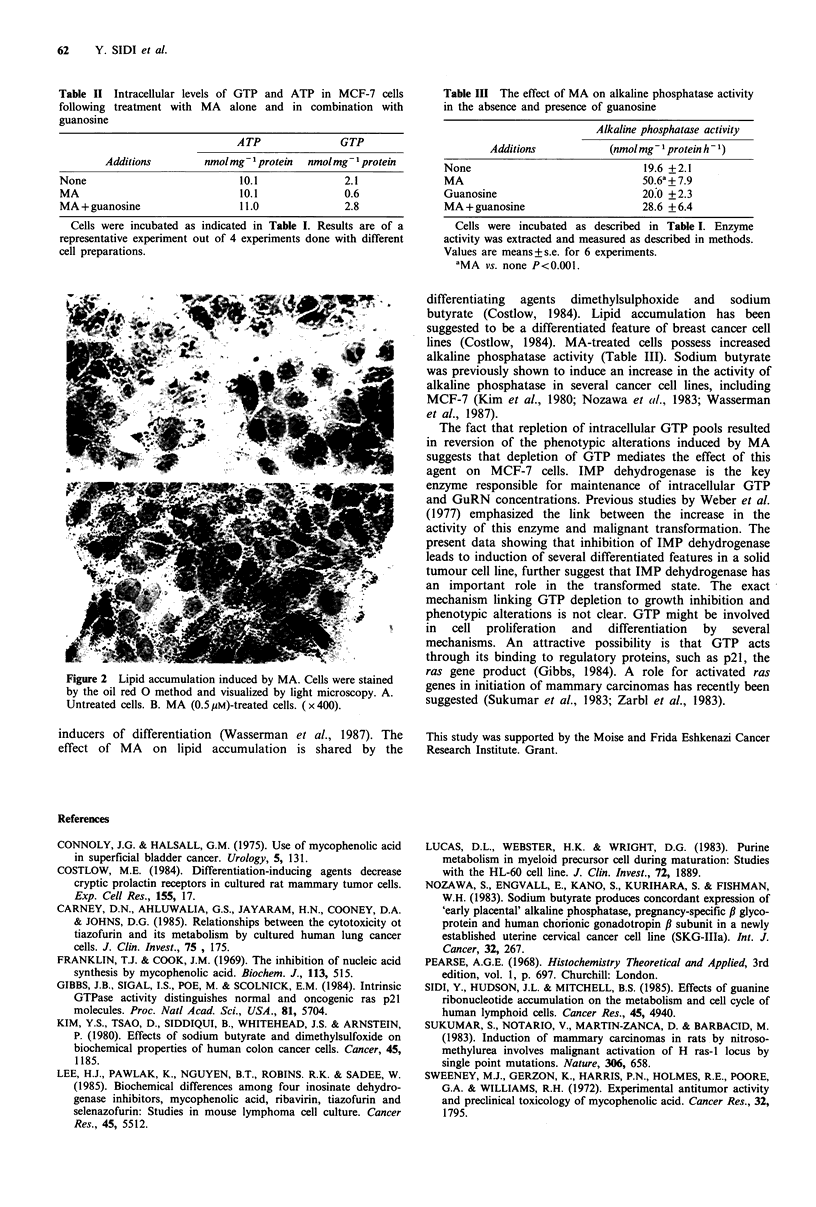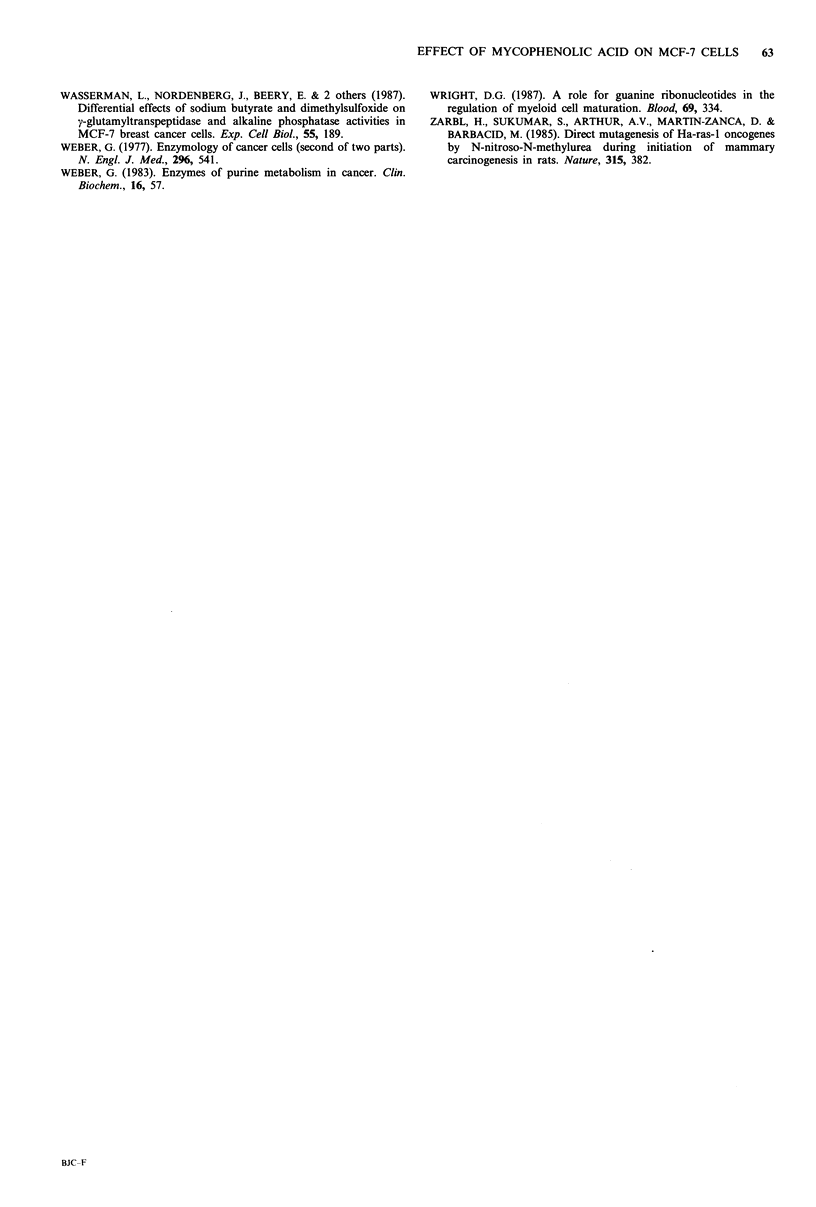# Growth inhibition and induction of phenotypic alterations in MCF-7 breast cancer cells by an IMP dehydrogenase inhibitor.

**DOI:** 10.1038/bjc.1988.162

**Published:** 1988-07

**Authors:** Y. Sidi, C. Panet, L. Wasserman, A. Cyjon, A. Novogrodsky, J. Nordenberg

**Affiliations:** Rogoff Medical Research Institute, Department of Medicine D, Beilinson Medical Center, Petah Tikva, Israel.

## Abstract

**Images:**


Br. J. Cancer (1988), 58, 61–63 © The Macmillan Press Ltd., 1988

SHORT COMMUNICATION

### Growth inhibition and induction of phenotypic alterations in MCF-7 breast cancer cells by an IMP dehydrogenase inhibitor

Y. Sidi[1,2], C. Panet[3], L. Wasserman[3], A. Cyjon[4], A. Novogrodsky[2,3] & J. Nordenberg[3]

[3]*The Rogoff Medical Research Institute, Departments of* [1]*Medicine D and* [4]*Oncology Department, Beilinson Medical Center, Petah Tikva 49100 and* [2]*Sackler School of Medicine, Tel-Aviv University, Israel.*

Guanine ribonucleotides (GuRN) play a major role in multiple cellular functions related to cell proliferation including DNA, RNA and protein synthesis (Weber, 1983). Intracellular GuRN concentration is mainly determined by the activity of inosinate (IMP) dehydrogenase and the availability of guanine for salvage by hypoxanthine guanine phosphoribosyl transferase (HGPRT). The activity of IMP dehydrogenase was shown to be markedly increased in transformed cells (Weber, 1977). Inhibitors of IMP dehydrogenase were found to be cytotoxic towards several tumour lines including those unresponsive to other chemotherapeutic agents (Sweeney *et al.*, 1972; Carney *et al.*, 1985; Connoly & Halsall, 1975). IMP dehydrogenase inhibitors have recently been shown to induce cell differentiation in the promyelocytic cell line HL-60 (Lucas *et al.*, 1983; Wright, 1987).

In the present study we examined the effects of mycophenolic acid (MA) on breast cancer cell lines. MA represents a prototypic IMP dehydrogenase inhibitor, with no other biochemical effects noted (Franklin & Cook, 1969; Lee *et al.*, 1985). Most studies were performed on the MCF-7 breast cancer cell line and selected experiments were done with the T-47D cell line. Cells were cultured as previously described (Wasserman *et al.*, 1987). For growth experiment cells ($10^5\,ml^{-1}$) were plated in culture medium (1.5 ml-RPMI 1640 supplemented with 10% foetal calf serum and antibiotics) in the absence and presence of MA. Seventy-two hours later the cells were detached with EDTA (1 mM) and counted in a Coulter counter.

The results depicted in Figure 1 show a marked dose dependent inhibitory effect of MA on MCF-7 breast cancer cell proliferation. Cell viability was not decreased by these concentrations of MA. Ninety-five per cent of cells were viable as assessed by the trypan blue dye exclusion test. The proliferation of the T-47D cell line was also inhibited by MA. Incubation of the cells with MA for 72 h at 0.5 $\mu$M decreased the number of the cells by 50%. It should be noted that B16 F10 mouse melanoma cells were also found to be highly sensitive to inhibition by MA (50% inhibition of cell growth was achieved by incubating cells for 48 h in the presence of 0.3 $\mu$M MA).

Exogenous addition of guanosine together with MA, or up to 6 h after MA reversed the anti-proliferative effect of MA on MCF-7 breast cancer cells (Table I). These results suggest that MA exerts its anti-proliferative effect via GTP depletion. The pools of intracellular GTP and ATP, following treatment of the cells with MA were determined by high liquid chromatography (Sidi *et al.*. 1985). It is shown that MA induces a marked depletion of GTP pools (Table II). Exogenous addition of guanosine restored GTP levels.

The anti-proliferative effect of MA was accompanied by phenotypic alterations. Lipid staining by the oil red O method (Pearse, 1968) revealed that MA induced lipid accumulation within the cells (Figure 2). The activity of the glycoprotein membrane-bound enzyme alkaline phosphatase was measured as previously described (Wasserman *et al.*, 1987) following incubation of the cells with MA. The results depicted in Table III show that MA markedly enhances the activity of this enzyme. The increase in alkaline phosphatase activity could be restored to that of untreated cells by the addition of exogenous guanosine.

The phenotypic changes induced by these compounds resemble those induced by agents known as chemical

**Figure 1** The effect of MA on MCF-7 cell growth. Cells ($1.5\times10^5$) were incubated (in 1.5 ml growth medium) with various concentrations of MA for 72 h. Values are means of 3 replicates.

**Table I** Reversion of the growth inhibitory effect of MA by addition of guanosine

| *Additions* | *Cell number* $\times 10^5$ *after 72 h incubation* |
|---|---|
| None | 6. 7 ± 0.7 |
| Guanosine | 7.2 ± 0.8 |
| MA | 3.4[a] ± 0.7 |
| MA + guanosine | 6.0 ± 0.9 |

$1.5\times10^5$ cells were cultured in the absence or presence of guanosine (400 $\mu$M) and MA (0.5 $\mu$M) for 72 h. Values are means ± s.e. of 5 experiments.
[a]MA *vs.* none $P<0.05$ MA + guanosine *vs.* MA $P<0.05$.

Correspondence: J. Nordenberg
Received 8 September 1987; and in revised form 18 February 1988.

**Table II** Intracellular levels of GTP and ATP in MCF-7 cells following treatment with MA alone and in combination with guanosine

| | *ATP* | *GTP* |
|---|---|---|
| *Additions* | *nmol mg$^{-1}$ protein* | *nmol mg$^{-1}$ protein* |
| None | 10.1 | 2.1 |
| MA | 10.1 | 0.6 |
| MA + guanosine | 11.0 | 2.8 |

Cells were incubated as indicated in **Table I**. Results are of a representative experiment out of 4 experiments done with different cell preparations.

**Table III** The effect of MA on alkaline phosphatase activity in the absence and presence of guanosine

| *Additions* | *Alkaline phosphatase activity (nmol mg$^{-1}$ protein h$^{-1}$)* |
|---|---|
| None | 19.6 ± 2.1 |
| MA | 50.6[a] ± 7.9 |
| Guanosine | 20.0 ± 2.3 |
| MA + guanosine | 28.6 ± 6.4 |

Cells were incubated as described in **Table I**. Enzyme activity was extracted and measured as described in methods. Values are means ± s.e. for 6 experiments.
[a]MA *vs.* none $P<0.001$.

**Figure 2** Lipid accumulation induced by MA. Cells were stained by the oil red O method and visualized by light microscopy. A. Untreated cells. B. MA (0.5 μM)-treated cells. (×400).

inducers of differentiation (Wasserman *et al.*, 1987). The effect of MA on lipid accumulation is shared by the differentiating agents dimethylsulphoxide and sodium butyrate (Costlow, 1984). Lipid accumulation has been suggested to be a differentiated feature of breast cancer cell lines (Costlow, 1984). MA-treated cells possess increased alkaline phosphatase activity (Table III). Sodium butyrate was previously shown to induce an increase in the activity of alkaline phosphatase in several cancer cell lines, including MCF-7 (Kim *et al.*, 1980; Nozawa *et al.*, 1983; Wasserman *et al.*, 1987).

The fact that repletion of intracellular GTP pools resulted in reversion of the phenotypic alterations induced by MA suggests that depletion of GTP mediates the effect of this agent on MCF-7 cells. IMP dehydrogenase is the key enzyme responsible for maintenance of intracellular GTP and GuRN concentrations. Previous studies by Weber *et al.* (1977) emphasized the link between the increase in the activity of this enzyme and malignant transformation. The present data showing that inhibition of IMP dehydrogenase leads to induction of several differentiated features in a solid tumour cell line, further suggest that IMP dehydrogenase has an important role in the transformed state. The exact mechanism linking GTP depletion to growth inhibition and phenotypic alterations is not clear. GTP might be involved in cell proliferation and differentiation by several mechanisms. An attractive possibility is that GTP acts through its binding to regulatory proteins, such as p21, the *ras* gene product (Gibbs, 1984). A role for activated *ras* genes in initiation of mammary carcinomas has recently been suggested (Sukumar *et al.*, 1983; Zarbl *et al.*, 1983).

This study was supported by the Moise and Frida Eshkenazi Cancer Research Institute. Grant.

### References

CONNOLY, J.G. & HALSALL, G.M. (1975). Use of mycophenolic acid in superficial bladder cancer. *Urology*, **5**, 131.

COSTLOW, M.E. (1984). Differentiation-inducing agents decrease cryptic prolactin receptors in cultured rat mammary tumor cells. *Exp. Cell Res.*, **155**, 17.

CARNEY, D.N., AHLUWALIA, G.S., JAYARAM, H.N., COONEY, D.A. & JOHNS, D.G. (1985). Relationships between the cytotoxicity ot tiazofurin and its metabolism by cultured human lung cancer cells. *J. Clin. Invest.*, **75** , 175.

FRANKLIN, T.J. & COOK, J.M. (1969). The inhibition of nucleic acid synthesis by mycophenolic acid. *Biochem. J.*, **113**, 515.

GIBBS, J.B., SIGAL, I.S., POE, M. & SCOLNICK, E.M. (1984). Intrinsic GTPase activity distinguishes normal and oncogenic ras p21 molecules. *Proc. Natl Acad. Sci., USA.*, **81**, 5704.

KIM, Y.S., TSAO, D., SIDDIQUI, B., WHITEHEAD, J.S. & ARNSTEIN, P. (1980). Effects of sodium butyrate and dimethylsulfoxide on biochemical properties of human colon cancer cells. *Cancer*, **45**, 1185.

LEE, H.J., PAWLAK, K., NGUYEN, B.T., ROBINS. R.K. & SADEE, W. (1985). Biochemical differences among four inosinate dehydrogenase inhibitors, mycophenolic acid, ribavirin, tiazofurin and selenazofurin: Studies in mouse lymphoma cell culture. *Cancer Res.*, **45**, 5512.

LUCAS, D.L., WEBSTER, H.K. & WRIGHT, D.G. (1983). Purine metabolism in myeloid precursor cell during maturation: Studies with the HL-60 cell line. *J. Clin. Invest.*, **72**, 1889.

NOZAWA, S., ENGVALL, E., KANO, S., KURIHARA, S. & FISHMAN, W.H. (1983). Sodium butyrate produces concordant expression of 'early placental' alkaline phosphatase, pregnancy-specific β glycoprotein and human chorionic gonadotropin β subunit in a newly established uterine cervical cancer cell line (SKG-IIIa). *Int. J. Cancer*, **32**, 267.

PEARSE, A.G.E. (1968). *Histochemistry Theoretical and Applied*, 3rd edition, vol. 1, p. 697. Churchill: London.

SIDI, Y., HUDSON, J.L. & MITCHELL, B.S. (1985). Effects of guanine ribonucleotide accumulation on the metabolism and cell cycle of human lymphoid cells. *Cancer Res.*, **45**, 4940.

SUKUMAR, S., NOTARIO, V., MARTIN-ZANCA, D. & BARBACID, M. (1983). Induction of mammary carcinomas in rats by nitrosomethylurea involves malignant activation of H ras-1 locus by single point mutations. *Nature*, **306**, 658.

SWEENEY, M.J., GERZON, K., HARRIS, P.N., HOLMES, R.E., POORE, G.A. & WILLIAMS, R.H. (1972). Experimental antitumor activity and preclinical toxicology of mycophenolic acid. *Cancer Res.*, **32**, 1795.

WASSERMAN, L., NORDENBERG, J., BEERY, E. & 2 others (1987). Differential effects of sodium butyrate and dimethylsulfoxide on γ-glutamyltranspeptidase and alkaline phosphatase activities in MCF-7 breast cancer cells. *Exp. Cell Biol.*, **55**, 189.

WEBER, G. (1977). Enzymology of cancer cells (second of two parts). *N. Engl. J. Med.*, **296**, 541.

WEBER, G. (1983). Enzymes of purine metabolism in cancer. *Clin. Biochem.*, **16**, 57.

WRIGHT, D.G. (1987). A role for guanine ribonucleotides in the regulation of myeloid cell maturation. *Blood*, **69**, 334.

ZARBL, H., SUKUMAR, S., ARTHUR, A.V., MARTIN-ZANCA, D. & BARBACID, M. (1985). Direct mutagenesis of Ha-ras-1 oncogenes by N-nitroso-N-methylurea during initiation of mammary carcinogenesis in rats. *Nature*, **315**, 382.